# Key learnings from the INBUILD trial in patients with progressive pulmonary fibrosis

**DOI:** 10.1177/17534666241266343

**Published:** 2024-08-07

**Authors:** Isabel Mira-Avendano, Mitchell Kaye

**Affiliations:** Department of Pulmonary, Critical Care and Sleep Medicine, UTHealth, Houston, TX 77030, USA; Minnesota Lung Center, Minneapolis, MN, USA

**Keywords:** clinical trial, disease progression, drug tolerance, lung disease, interstitial, pulmonary fibrosis

## Abstract

In a patient with interstitial lung disease (ILD) of known or unknown etiology other than idiopathic pulmonary fibrosis (IPF), progressive pulmonary fibrosis (PPF) is defined by worsening lung fibrosis on high-resolution computed tomography (HRCT), decline in lung function, and/or deterioration in symptoms. The INBUILD trial involved 663 patients with PPF who were randomized to receive nintedanib or placebo. The median exposure to trial medication was approximately 19 months. The INBUILD trial provided valuable learnings about the course of PPF and the efficacy and safety of nintedanib. The relative effect of nintedanib on reducing the rate of forced vital capacity decline was consistent across subgroups based on ILD diagnosis, HRCT pattern, and disease severity at baseline, and between patients who were and were not taking glucocorticoids or disease-modifying anti-rheumatic drugs and/or glucocorticoids at baseline. The adverse events most frequently associated with nintedanib were gastrointestinal, particularly diarrhea, but nintedanib was discontinued in only a minority of cases. The results of the INBUILD trial highlight the importance of prompt detection and treatment of PPF and the utility of nintedanib as a treatment option.

## Plain language summary

What did we find out from the INBUILD trial about progressive lung fibrosis?

Lung fibrosis is a rare disease in which the lung tissue becomes scarred and hardened. This makes it more difficult for the lungs to inflate and for the lungs to exchange oxygen with the blood. In some patients, lung fibrosis gets worse over time. This is known as progressive lung fibrosis. In the INBUILD trial, researchers looked at the effects of a drug called nintedanib in patients with progressive lung fibrosis. In this trial, 663 patients were randomly allocated to receive either nintedanib or a placebo and then followed for approximately 19 months. The patients and the researchers did not know which patients were taking the active drug (nintedanib) and which patients were taking placebo. The results showed that the criteria used to find patients with progressive lung fibrosis to take part in the trial successfully identified patients whose disease would continue to worsen. These criteria were based on a decline in the volume (size) of the lungs, worsening symptoms such as shortness of breath, and worsening of changes seen on a scan of the chest. The trial results also showed that nintedanib slowed down loss of lung function and had a similar benefit in patients with different severities of disease at the start of the trial. The most common side-effects of nintedanib were gastrointestinal problems, particularly diarrhea, but most patients given nintedanib were able to cope with these side-effects without needing to stop treatment. Large trials like the INBUILD trial are important for helping us understand how diseases progress and in which patients particular drugs should be used.

## Introduction

The INBUILD trial was a randomized placebo-controlled trial of nintedanib in patients with progressive fibrosing interstitial lung diseases (ILDs) other than idiopathic pulmonary fibrosis (IPF).^1^ This trial enrolled patients who met one of the following criteria for progression of ILD at any time within the prior 2 years, despite management in clinical practice: relative decline in forced vital capacity (FVC) % predicted ⩾10%; relative decline in FVC % predicted ⩾5% to <10% and worsened respiratory symptoms; relative decline in FVC % predicted ⩾5% to <10% and increased extent of fibrosis on high-resolution computed tomography (HRCT); and worsened respiratory symptoms and increased extent of fibrosis on HRCT. These differ from the criteria for progressive pulmonary fibrosis (PPF) that were later proposed by international respiratory societies, which included worsening respiratory symptoms and an absolute decline in FVC % predicted ⩾5% or absolute decline in DLCO % predicted ⩾10%, and used a 1-year rather than 2-year time window.^2^ A relative decline in FVC % predicted of >10% is the strongest predictor of transplant-free survival in patients with fibrosing ILDs.^3^

In the INBUILD trial, patients were randomized to receive nintedanib or placebo, stratified by whether they had a usual interstitial pneumonia (UIP)-like fibrotic pattern or other fibrotic patterns on HRCT. The primary endpoint was the rate of decline in FVC (mL/year) assessed over 52 weeks. The median follow-up in the trial was approximately 19 months. As well as providing robust data on the efficacy and safety of nintedanib in this patient population, the findings of the INBUILD trial provided valuable information on the clinical course of progressive lung fibrosis.

## Key learnings from the INBUILD trial

### Learning 1: Patients with PPF can be identified in clinical practice

**Figure 1.**
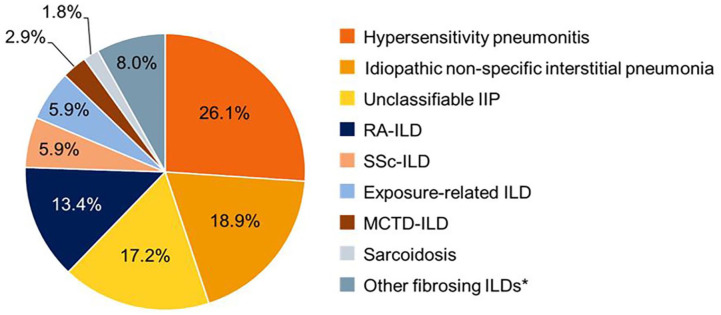
ILD diagnoses in the INBUILD trial. *Source*: Wells et al.^4^ Copyright (2020), with permission from Elsevier. Included other autoimmune lLDs (e.g. Sjögren’s syndrome-associated ILD, systemic lupus erythematosus-associated ILD), and other llPs (e.g. pleuroparenchymal fibroelastosis, cryptogenic organizing pneumonia, desquamative interstitial pneumonia). IIP, idiopathic interstitial pneumonia; ILDs, interstitial lung diseases; MCTD, mixed connective tissue disease; RA, rheumatoid arthritis; SSc, systemic sclerosis.

**Figure 2.**
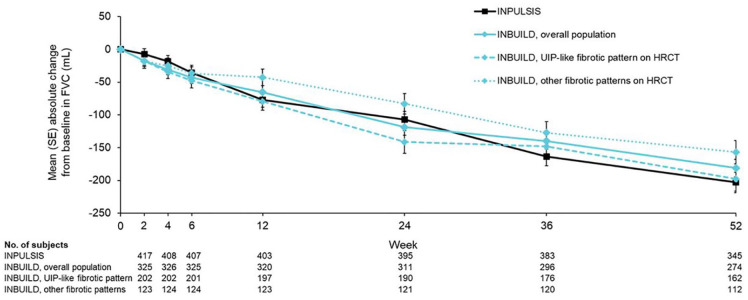
Course of FVC decline in the INPULSIS trials in patients with IPF and the INBUILD trial in patients with progressive fibrosing ILDs other than IPF.^8^ *Source*: Brown et al.^8^ Reproduced with permission of the © ERS 2023. IPF, idiopathic pulmonary fibrosis; ILDs, interstitial lung diseases.

The INBUILD trial enrolled 663 patients with a variety of ILD diagnoses (Figure 1),^4^ of whom 62% had a UIP-like fibrotic pattern on HRCT.^1^ While different criteria have been proposed for the identification of PPF,^2,5–7^ there is no doubt that the inclusion criteria used in the INBUILD trial identified patients with progressive disease: over 52 weeks, FVC declined by a mean of 193 mL in the patients who received placebo, similar to the mean decline of 221 mL observed in the placebo group of the INPULSIS trials in patients with IPF (Figure 2).^1,8^ In the placebo group of the INBUILD trial, the rate of decline in FVC was similar across the subgroups by ILD diagnosis.^8^ There was a notable decline in FVC across all subgroups based on which of the inclusion criteria for ILD progression the patients fulfilled, but the decline was greatest among those who met the criterion of a decline in FVC % predicted ⩾10%.^9^ Patients with a UIP-like fibrotic pattern on HRCT showed a faster decline in FVC than those with other fibrotic patterns, but there was a marked decline in FVC over 52 weeks in both these subgroups (215 and 160 mL in the placebo group).^8^ Over a median follow-up of approximately 19 months, 8.7% of patients experienced an acute exacerbation of ILD; the risk of mortality in the 180 days following an acute exacerbation was 37%.^10^

### Learning 2: Nintedanib slows the progression of pulmonary fibrosis

**Figure 3.**
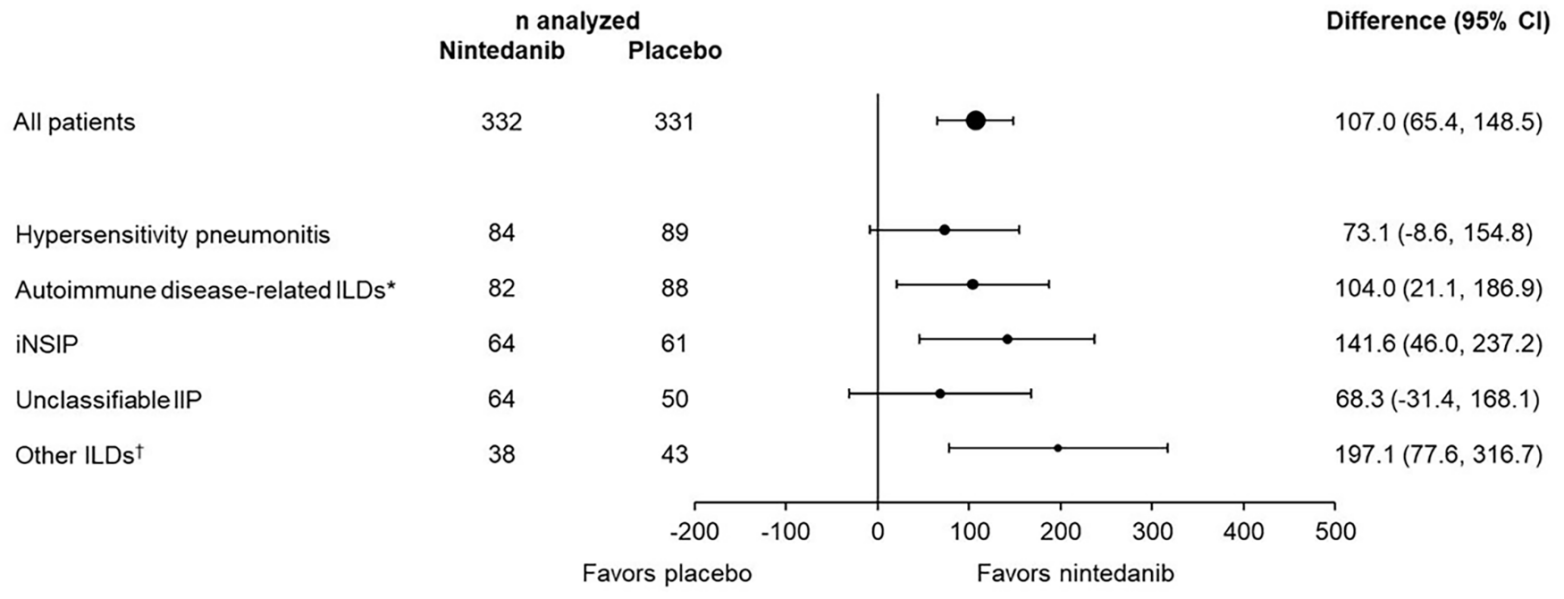
Consistent effect of nintedanib on reducing the rate of decline in FVC across subgroups by ILD diagnosis in the INBUILD trial.^4^ *Source*: Wells et al.^4^ Copyright (2020), with permission from Elsevier. Treatment-by-subgroup-by-time interaction *p* = 0.41. *Included rheumatoid arthritis-associated ILD, systemic sclerosis-associated ILD, mixed connective tissue disease-associated ILD, plus autoimmune disease-related ILDs in the “other fibrosing ILDs” category of the case report form. ^†^Included sarcoidosis, exposure-related ILDs, and other terms in the “other fibrosing ILDs” category of the case report form. FVC, forced vital capacity; ILD, interstitial lung disease.

In the INBUILD trial, the adjusted rate of decline in FVC over 52 weeks was 80.8 mL/year in the nintedanib group and 187.8 mL/year in the placebo group (difference: 107.0 (95% CI: 65.4, 148.5); *p* < 0.001).^1^ In patients with a UIP-like fibrotic pattern on HRCT, the adjusted rate of decline in FVC was 82.9 mL/year with nintedanib and 211.1 mL/year with placebo (difference: 128.2 (95% CI: 70.8, 185.6)) while in patients with other fibrotic patterns on HRCT, the adjusted rate of decline in FVC was 79.0 mL/year with nintedanib and 154.2 mL/year with placebo (difference: 75.3 (95% CI: 15.5, 135.0)).^1^ The relative effect of nintedanib on reducing the rate of FVC decline was consistent across subgroups based on ILD diagnosis (Figure 3),^4,11^ supporting the concept of “lumping” patients with progressive lung fibrosis, irrespective of etiology, when studying the efficacy of an intervention.^12,13^ The effect of nintedanib on slowing decline in FVC was also consistent across subgroups by disease severity at baseline, based on FVC % predicted or the GAP stage.^14^ Treatment with nintedanib reduced the risk of an acute exacerbation of ILD: over the whole trial, the proportions of patients who had an acute exacerbation or died were 13.9% in the nintedanib group and 19.6% in the placebo group (HR 0.67 (95% CI: 0.46, 0.98)).^15^ The INBUILD trial was not powered to show a significant difference in survival between the treatment groups, but estimates of long-term survival based on a Bayesian borrowing approach suggest that nintedanib prolongs survival in this patient population by 2.6–3.0 years.^16^

### Learning 3: Nintedanib might be used first-line or as an add-on therapy

The inclusion criteria in the INBUILD trial specified that patients’ ILD must have progressed despite management deemed appropriate in clinical practice, but did not mandate that particular therapies, or indeed any therapies, must have been used prior to a patient being enrolled. Most of the patients in the INBUILD trial were taking immunosuppressants. At baseline, 53.2% of patients were taking low-dose glucocorticoids (⩽20 mg/day prednisone or equivalent), 4.7% were taking biologic disease-modifying antirheumatic drugs (DMARDs), and 11.6% were taking non-biologic DMARDs.^17^ In subgroup analyses, nintedanib had a consistent effect on reducing the rate of decline in FVC in patients who were and were not taking glucocorticoids, or DMARDs and/or glucocorticoids, at baseline.^14,17^ The introduction of immunomodulatory therapies during the trial did not affect the benefit of nintedanib in reducing the rate of FVC decline.^17^ Safety data from the INBUILD trial,^17,18^ and from the SENSCIS trial in patients with systemic sclerosis-associated ILD,^19^ suggest that immunomodulatory therapies and nintedanib have acceptable tolerability when given together. It should be noted that data from the INBUILD and SENSCIS trials cannot be used to determine the efficacy of immunomodulatory therapies in the treatment of ILD, as the patients were not randomized by the use of these therapies. Nor can the data from this trial be used to determine what therapies should be “failed” before nintedanib is initiated. The latest international guidelines for the management of patients with PPF provide a conditional recommendation for the use of nintedanib in patients who have failed standard management for fibrotic ILD, but do not specify what standard management should entail or how treatment failure should be defined.^2^

While data were not collected on the therapies that patients received before being enrolled in the INBUILD trial, not all patients were taking immunosuppressants at baseline, suggesting that in some patients, nintedanib may have been used as first-line therapy. Immunosuppression has not been shown to be beneficial in the treatment of all forms of PPF; for example, there is little evidence of a long-term benefit of immunosuppression in patients with fibrotic hypersensitivity pneumonitis^20,21^ and no evidence of efficacy in patients with other exposure-related ILDs. For patients with hypersensitivity pneumonitis or exposure-related ILDs, removal of the culprit exposure should be first-line management. Immunosuppression should not be used as a chronic treatment for IPF, as it is harmful in these patients.^22^ These data suggest the potential for the use of nintedanib as a first-line treatment for PPF, but the efficacy of nintedanib in drug-naïve patients with PPF remains to be studied.

### Learning 4: The side effects of nintedanib therapy need to be managed

**Figure 4.**
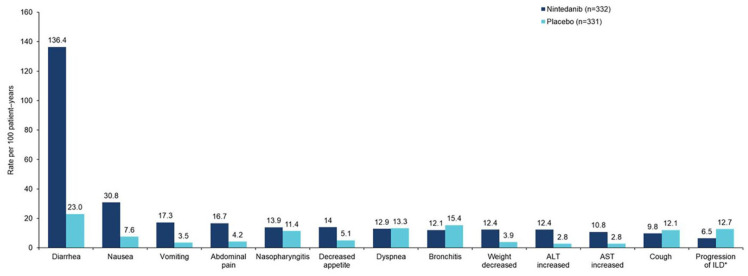
Most frequent adverse events reported in the INBUILD trial.^18^ Data are based on adverse events reported between the first trial drug intake and 28 days after the last trial drug intake. The median exposure to the trial drug was 17.4 months in both groups. Adverse events were coded based on single preferred terms in the Medical Dictionary for Regulatory Activities (MedDRA) version 22.0, except for abdominal pain, which was based on a group of MedDRA-preferred terms. Adverse events with a rate >10 events per 100 patient-years in either treatment group are shown. *Based on MedDRA preferred term interstitial lung disease. ALT, alanine aminotransferase; AST, aspartate aminotransferase.

As observed in other clinical trials and real-world studies,^18,23–25^ the adverse events most frequently associated with nintedanib in the INBUILD trial were gastrointestinal events, particularly diarrhea (Figure 4). Recommendations for prevention and management of the side effects that may occur in patients receiving nintedanib were provided in clinical trials and can also be applied in clinical practice. These include taking the drug with meals, the use of symptomatic therapies (e.g. loperamide), dose reduction from 150 mg twice daily to 100 mg twice daily, and/or treatment interruption if side effects occur.^18,23,24^ Over a median follow-up of 17.4 months, 48.2% and 15.7% of patients had at least one dose reduction and/or treatment interruption, and adverse events led to permanent treatment discontinuation in 22.0% and 14.5% of patients, in the nintedanib and placebo groups, respectively.^18^ Diarrhea was reported in 72.3% of patients treated with nintedanib and 25.7% of patients who received placebo, but led to treatment discontinuation in only 6.3% and 0.3% of the nintedanib and placebo groups, respectively.^18^

Many patients with pulmonary fibrosis experience weight loss.^26^ Nintedanib is associated with an increased risk of weight loss. Over the whole INBUILD trial, the decrease in weight associated with treatment with nintedanib versus placebo was about 2 kg.^27^ A lower body mass index at baseline and weight loss during the trial were associated with an increased risk of acute exacerbation or death.^27^ Clinicians should monitor weight in patients treated with nintedanib and suggest interventions to maintain/increase weight if required.

Nintedanib is associated with a risk of liver enzyme elevations.^18,23,24^ It is recommended that liver function tests should be conducted prior to initiation of the therapy, at regular intervals during the first 3 months of treatment, and periodically thereafter. For most cases of liver enzyme elevations in the INBUILD trial, levels returned to within the normal range spontaneously or after nintedanib dose reduction or interruption.^18^ As an inhibitor of the vascular endothelial growth factor receptor, nintedanib may increase the risk of bleeding.^28^ In the INBUILD trial, bleeding adverse events were not more frequent in patients treated with nintedanib than placebo, but it should be noted that patients treated with full-dose anticoagulation, high-dose antiplatelet therapy, or at high risk of bleeding were excluded from the trial.^18^

The adverse event profile of nintedanib in the INBUILD trial was generally consistent across subgroups based on age, sex, race, and weight, but nausea, vomiting, and dose reductions were more common among female than male patients.^18,29^ It is not possible to predict which patients given nintedanib will develop particular side effects and all patients should be counseled on how to manage them if they occur. Among patients who completed 52 weeks of treatment in the INBUILD trial, the rate of decline in FVC was similar irrespective of dose adjustments used to manage adverse events^18^; however, these data should not be misinterpreted as indicating that patients should be started or maintained on the lower dose. Data from the open-label extension of the INBUILD trial, INBUILD-ON, indicate that the safety and tolerability profile of nintedanib is maintained over longer-term use.^30^

### Learning 5: Patients with PPF should be identified and treated promptly

Community pulmonologists have a key role to play in the identification of patients with pulmonary fibrosis and in detecting and monitoring progression. The diagnosis of ILD and the detection of disease progression are often delayed.^31^ To date, no specific guidelines for monitoring patients with fibrosing ILDs have been issued by a professional organization. However, experts in the field have recommended that patients with ILD should be monitored for disease progression *via* regular pulmonary function tests, review of symptoms, and repeat HRCT scans (where appropriate).^32–36^ Patients with worsening pulmonary function tests or symptoms require a thorough clinical assessment, as these may worsen for reasons other than progression of ILD.

An individualized approach to the treatment of pulmonary fibrosis is required, taking into consideration disease severity, evidence of disease progression, risk factors for progression, comorbidities, health-related quality of life, and the preferences of the patient. Initial management of pulmonary fibrosis should be tailored to the type of ILD. In patients whose pulmonary fibrosis progresses, prompt treatment is needed to slow progression, as fibrosis is a self-sustaining process,^37,38^ and lung that is lost to fibrosis cannot be recovered. Furthermore, data from the INBUILD trial^39^ as well as other studies in patients with PPF^40,41^ have shown that deterioration in lung function is associated with short-term mortality. Patients with pulmonary fibrosis need information on their disease, prognosis, and treatment options, including non-pharmacological strategies such as pulmonary rehabilitation and supportive/palliative care, so that they can make informed decisions about their care.^42^

## Conclusion

The INBUILD trial provided criteria that can be used to identify patients with PPF in clinical practice and valuable information on the course and impact of PPF. The results of this trial highlight the importance of prompt detection and treatment of PPF and the efficacy and safety of nintedanib as a possible treatment option. Additional clinical trials and observational studies will provide further evidence to inform best practice in the identification, monitoring, and treatment of PPF.

A video abstract summarizing the contents of this article is available at: https://www.usscicomms.com/respiratory/LearningsFromINBUILDTrial

## Declarations

**Ethics approval and consent to participate:** Not applicable.

**Consent for publication:** Not applicable.

**Availability of data and materials:** Not applicable.
